# Impact of CYP2B6 and CYP2A6 polymorphisms on efavirenz plasma concentrations in Ghanaian HIV-infected patients

**DOI:** 10.1186/1758-2652-13-S4-P183

**Published:** 2010-11-08

**Authors:** Y Zhang, A Owen, D Egan, SF Sarfo

**Affiliations:** 1 University of Liverpool, Department of Pharmacology and Therapeutics, Liverpool, UK; 2 Komfo Anokye Teaching Hospital, Kumasi, Ghana

## Purpose of the study

Genetic variations in the enzymes responsible for the metabolism of efavirenz partially explain inter-individual variability in serum efavirenz concentrations. In this cross-sectional study we determined the frequency and impact on efavirenz plasma concentrations of the CYP2B6 516G>T and CYP2A6*9 polymorphisms.

## Methods

Following informed consent, blood samples were obtained from 521 adults on efavirenz based ART. Drug concentrations approximately 12-14h post-dose were measured using a validated HPLC with UV detection. Total genomic DNA was extracted by standard methodology and patients were genotyped using real-time PCR with allelic discrimination.

## Results

The frequency of CYP2B6 516G>T and CYP2A6*9 genotypes were GG 29.8%, GT 44.3%, TT 25.9% and CC 91.6%, AC 8.7%, and AA 0.39%, respectively. Both polymorphisms were statistically associated with efavirenz plasma concentrations. Median plasma concentrations according to CYP2B6 516G>T were 1297, 1833 (P < 0.05) and 2248 (P < 0.001) µg/ml for GG, GT and TT individuals, respectively. Median plasma concentrations according to CYP2A6*9 were 1713, 3225 (P < 0.0001), and 1231 µg/ml for CC, CA and AA individuals, respectively. Median efavirenz concentrations in 268 males were 1759 µg/ml and in 253 females were 1826µg/ml (P > 0.05).

## Conclusions

Our results show that polymorphisms in CYP2B6 and CYP2A6 genes are significantly associated with plasma concentrations of efavirenz in Ghanaian HIV infected patients. Further studies are now warranted to explore the potential for pharmacogenetics-directed dose individualisation of efavirenz. A prospective phase of the study is in progress to evaluate the influence of genetic polymorphisms on efavirenz concentrations and CNS toxicity.

